# Identifying Thresholds for Ecosystem-Based Management

**DOI:** 10.1371/journal.pone.0008907

**Published:** 2010-01-26

**Authors:** Jameal F. Samhouri, Phillip S. Levin, Cameron H. Ainsworth

**Affiliations:** 1 Pacific States Marine Fisheries Commission, Portland, Oregon, United States of America; 2 National Oceanic and Atmospheric Administration, National Marine Fisheries Service, Northwest Fisheries Science Center, Seattle, Washington, United States of America; 3 Marine Resources Assessment Group Americas Inc., St. Petersburg, Florida, United States of America; NOAA/NMFS/SWFSC, United States of America

## Abstract

**Background:**

One of the greatest obstacles to moving ecosystem-based management (EBM) from concept to practice is the lack of a systematic approach to defining ecosystem-level decision criteria, or reference points that trigger management action.

**Methodology/Principal Findings:**

To assist resource managers and policymakers in developing EBM decision criteria, we introduce a quantitative, transferable method for identifying utility thresholds. A utility threshold is the level of human-induced pressure (e.g., pollution) at which small changes produce substantial improvements toward the EBM goal of protecting an ecosystem's structural (e.g., diversity) and functional (e.g., resilience) attributes. The analytical approach is based on the detection of nonlinearities in relationships between ecosystem attributes and pressures. We illustrate the method with a hypothetical case study of (1) fishing and (2) nearshore habitat pressure using an empirically-validated marine ecosystem model for British Columbia, Canada, and derive numerical threshold values in terms of the density of two empirically-tractable indicator groups, sablefish and jellyfish. We also describe how to incorporate uncertainty into the estimation of utility thresholds and highlight their value in the context of understanding EBM trade-offs.

**Conclusions/Significance:**

For any policy scenario, an understanding of utility thresholds provides insight into the amount and type of management intervention required to make significant progress toward improved ecosystem structure and function. The approach outlined in this paper can be applied in the context of single or multiple human-induced pressures, to any marine, freshwater, or terrestrial ecosystem, and should facilitate more effective management.

## Introduction

Ecosystem-based management (EBM) has moved to the forefront of efforts to conserve and restore marine species and ocean ecosystems. Implementing EBM requires quantitative methods and criteria that can be used to assess overall ecosystem status, evaluate trade-offs among ecosystem services, and guide management actions [1]. However, the science of EBM is young relative to that of single-species management. Practitioners of single-species management set decision criteria based on well-vetted stock assessment models [2] and population viability analysis methods [3], among other approaches. These decision criteria are measureable quantities, defined in terms of species' attributes (e.g., abundance, size-structure) or human-induced pressures (e.g., fishing yields or rates), intended to prompt management action. Analogous decision criteria with a broader focus on community- and ecosystem-level attributes and pressures are only in a nascent stage of development [4]–[6].

In the context of EBM, a decision criterion is most sensibly set according to (1) scientific understanding of ecosystem dynamics, and in particular of ecological thresholds, and (2) the desired biological, chemical, and physical states and functions (or processes) in the ecosystem. An ecological threshold is a point at which small changes in environmental conditions produce large, and sometimes abrupt, responses in ecosystem state or function [7]. A classic example of an ecological threshold comes from studies of freshwater lakes: beyond a critical level of nutrient input, a clear-water lake can become turbid and dominated by phytoplankton blooms [8]. In a management context, the environmental conditions relevant to ecological thresholds will often be some type of natural (e.g., grazing rate, upwelling intensity) or anthropogenic (e.g., pollution, harvest) pressure.

Desired ecosystem states and functions are typically based on value judgments of stakeholders, and can be used to define utility thresholds. A utility threshold is a point at which small changes in environmental conditions produce substantial improvements in the management outcome [9]. Following the freshwater lake example, if the desired ecosystem state is characterized by clear water, and small reductions in nutrient input at the ecological threshold produce large increases in water clarity, then the utility threshold may coincide with the ecological threshold. Note, however, that the existence of an ecological threshold is not a prerequisite for the identification of a utility threshold. Rather, the requirement for a utility threshold is only that the ecosystem response to environmental conditions be nonlinear, so that some management actions produce greater changes in ecosystem states or functions than others (cf. [6]). Policymakers may choose to set the decision criterion, or trigger for management action, at a more conservative point than the utility threshold in order to reduce risk [10]. Thus in the freshwater lake example the decision criterion might correspond to a lower level of nutrient input than the utility threshold.

In this paper, we introduce a quantitative method for identifying utility thresholds. We describe how to incorporate uncertainty into the estimation of utility thresholds, demonstrate how utility thresholds can be translated into empirically-tractable metrics, and highlight the value of utility thresholds for understanding trade-offs among management objectives within an EBM context. To illustrate the analytical methods, we focus our presentation on two hypothetical case studies of (1) fishing and (2) nearshore habitat pressures using a marine ecosystem model for British Columbia, Canada.

## Methods

### Quantitative Identification of Utility Thresholds

The first step in a utility threshold analysis is to specify the EBM objectives in terms of ecosystem attributes and human-induced pressures. We define ecosystem attributes as aspects of ecosystem state and function relevant to the EBM goal of maintaining a healthy, productive, and resilient ecosystem [11]. For instance, attributes such as ecosystem energetics, nutrient cycling rates, and a variety of community structure metrics (e.g., diversity, food chain length, etc.) have deep theoretical or empirical underpinnings [12]–[18] and will be important in nearly all management contexts. In order to proceed with the utility threshold analysis, the values of the ecosystem attributes under stressed and unstressed conditions (sensu [13]) should be known, at least qualitatively.

Human-induced pressures (*sensu* [19]) are direct stressors that affect the natural environment. These include activities such as pollution, harvest, and habitat alteration, among many others. It is critical that specific pressures and a possible range of values for them are selected so that potentially different responses of (1) a single ecosystem attribute to alternative pressures, or (2) several ecosystem attributes to a single pressure, can be quantified explicitly. The selection of ecosystem attributes and human-induced pressures on which to focus will be based largely on value judgments of stakeholders (cf. [20], [21]), but it is nonetheless an essential step toward constraining the analysis that follows.

**Figure 1:**
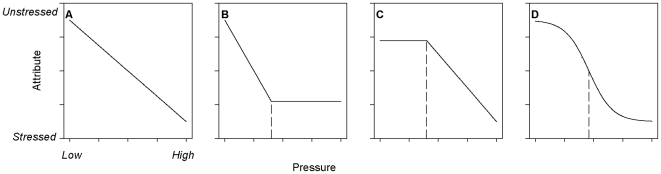
Relationships between hypothetical ecosystem attributes and anthropogenic pressures. Attribute values range from unstressed to stressed (sensu [13]), and the levels of the pressures applied have been scaled relative to a theoretical maximum. A utility threshold cannot be defined objectively for the linear model (a), but can be defined objectively for the two piecewise models (b and c) and the sigmoidal model (d). Equations for the models and the location of the utility thresholds are described in Text S1. In (b-d), the threshold pressure is indicated by the dashed lines.

The second step in the utility threshold analysis is to determine the relationship between the ecosystem attributes and the pressure(s) of management concern. This step can be conducted using empirical data and/or quantitative models, depending on their availability. Empirical understanding of attribute-pressure relationships based on spatial correlations, time series, and experiments (e.g., [5], [8], [22]), are valuable sources of this information. Alternatively, models can be used to understand the effects of increasing pressure(s) on specific ecosystem attributes (e.g., [23]). The shape of the relationship between an attribute that responds to a pressure is likely to resemble qualitatively one of the four schematics shown in Fig. 1 [5], [24].

The relationships between each ecosystem attribute and pressure should be established formally by confronting the data (or model output) with alternative mathematical functions and performing a model selection analysis. The goal of this analysis is to distinguish linear (Fig. 1a) from nonlinear (Figs. 1b-d) attribute-pressure relationships. The shape of the attribute-pressure relationship and the ease with which an objective threshold point can be defined should drive the choice of which mathematical functions to consider in the model selection analysis. In Fig. 1, the linear (Fig. 1a), concave-up piecewise (Fig. 1b), concave-down piecewise (Fig. 1c), and sigmoidal (Fig. 1d) relationships can be represented by simple functions with well-known mathematical properties (Text S1).

The third step in this analysis is to identify the utility threshold in any attribute-pressure relationship judged to be nonlinear. Here we discuss the conceptual basis for the location of the threshold point; further details regarding the mathematical definitions can be found in Text S1. We propose that a nonlinear relationship allows the identification of a utility threshold because the value of the attribute changes rapidly with declines in pressure over a specific region of parameter space, but changes more slowly elsewhere. Thus, for a nonlinear attribute-pressure relationship the potential gains toward achieving EBM goals by reducing a pressure are greatest where the curve is steepest. Mathematically, this definition is focused on the derivatives of the nonlinear functions.

In the case of the piecewise model, the threshold point *P_t_* is obtained when statistically-fitting the model to the data, and represents the intersection of two lines, each with a different slope (or first derivative; Figs. 1b-c). Modifying the pressure in the region where the absolute value of the slope is large will produce correspondingly large changes in the value of the attribute, whereas modifying the pressure elsewhere will have a lesser effect. Because it allows an objective definition of the utility threshold, the piecewise model can serve as a convenient approximation for curvilinear attribute-pressure relationships (resembling Figs. 1b-c) that continuously accelerate or decelerate (i.e., the second derivative is never equal to zero). Similar piecewise functions have been used widely to identify thresholds for biological effects of contaminants [25], edge effects on ecological communities [26], phase-dependent dynamics in cyclic populations [27], and density-dependent mortality in fish populations [28], to name just a few examples.

In the case of the sigmoidal model, we define the utility threshold at the point where small changes in the pressure have the greatest influence on the value of the attribute (i.e., where the first derivative is minimized) and where the function switches from concave-down to concave-up (i.e., where the second derivative is equal to zero). Modifying the amount of pressure near the threshold (Fig. 1d) will produce much larger changes in the value of the attribute than will adjustments elsewhere. Theoretical discussions of ecological thresholds commonly refer to this type of attribute-pressure relationship, with the idea that the attribute will tend to diminish with increases in pressure above the threshold and will tend to rise with reductions in pressure below the threshold [29], [30].

We have defined utility thresholds in terms of pressures and attributes, but in practice, it may be difficult to measure the amount of pressure or the value of the attribute on the scale of the entire ecosystem. The fourth step in the utility threshold analysis is to translate the threshold pressure in any nonlinear attribute-pressure relationship to values of easily-measured indicators representative of the status or trend in the ecosystem attribute. The performance and reliability of candidate indicators can be tested via empirical studies (e.g., time series, spatial contrasts) or model simulation [18], [31]–[34]. Each utility threshold should be translated into a value for multiple reliable indicators, and empirical analysis should be used to judge the current status of the indicators relative to the corresponding utility threshold values. A consensus among indicators that a utility threshold has or has not been breached can in turn inform management decisions.

### Case Studies

We present two hypothetical case studies, one related to fishing pressure and the other to pressure associated with changing nearshore habitat quality, using an empirically-validated marine ecosystem model for northern British Columbia, Canada (2000 AD; [35]). This Ecopath with Ecosim (EwE) model consists of 53 trophically-linked functional groups (including 4 marine mammal, 32 fish, 12 invertebrate, 1 seabird, 2 primary producer, and 2 detritus groups). Its dynamics are determined by specified predator-prey relationships, recruitment processes, fishing rates, and physical forcing that allow numerical simulation of a mass-balanced trophic model (for details about EwE, see [36]).

For the sake of illustration, we focus the utility threshold analysis on four ecosystem attributes: resilience (defined below), the ratio of net primary production (NPP) to total ecosystem biomass, Shannon diversity, and mean trophic level (Table 1). (We also chose these ecosystem attributes because we could determine their sensitivity to model parameters using the available software). Following Samhouri et al. [34], we measured resilience as the degree of biomass reorganization in the ecosystem following a perturbation. Specifically, the index *R* describes the difference in biomass abundances of individual functional groups (*B_t,i_*) prior to (*t_1_*) and following (*t_2_*) the application of a pressure. These differences are expressed relative to the difference in the aggregate biomass of all functional groups before and after the pressure is applied:
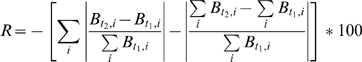
(1)


**Table 1:** Ecosystem attributes measured in simulations of increasing fishing and nearshore habitat pressure in the Northern British Columbia Ecopath with Ecosim marine food web model.

Attribute	Definition	Reference
Resilience	The capacity of an ecosystem to absorb perturbations while retaining its essential structure and function, including the identities of the component species. (see equation 1)	[16]
NPP / Biomass	The ratio of net primary production (NPP) to total biomass (less detritus and fishery discards) in the ecosystem; a measure of ecosystem maintenance costs.	[13]
Shannon diversity	Both the number of species and the evenness of biomass distribution among species.	[13], [14]
Mean trophic level	Biomass-weighted average trophic level of all species in the ecosystem.	[43], [63]

A smaller value of *R* (i.e., farther from zero) indicates lower resilience because it implies that the aggregate biomass and the individual functional groups responded differently in magnitude and direction to a pressure.

Understanding how ecosystem attributes such as these change in response to increasing anthropogenic pressure can be useful for examining trade-offs implied by alternative management scenarios. We explored trade-offs among the four attributes and simultaneously considered two ecosystem service metrics related to fisheries yield: total landings and total market value. Total market value was calculated by using the modal gear-specific market price for each functional group [35], and assumed constant across varying levels of anthropogenic pressure. To facilitate comparison of attribute-pressure relationships, we re-scaled the attributes (so that small values indicated a stressed condition and large values indicated an unstressed condition) and standardized them to zero mean and unit variance. We also re-scaled the range of pressures so that the lowest stress was indicated by zero and the highest stress by one.

It is impossible to account comprehensively for all potential sources of uncertainty in an ecosystem model of this complexity [37]. However, we demonstrate a method to account for uncertainty that can be applied to a subset of model parameters for which estimates are believed to be least reliable or for which ecosystem responses are most sensitive. Specifically, we included probability distributions for four parameters (biomass, production/biomass, ecotrophic efficiency, and biomass accumulation rate; see [36] for parameter definitions) related to each of the six benthic invertebrate groups in the model (large crabs, small crabs, commercial shrimp, epifaunal invertebrates, infaunal carnivorous invertebrates, and infaunal invertebrate detritivores), because in many systems these data are least reliable and least abundant. We used the EwE Monte Carlo resampling routine (*n* = 100 simulations) to generate a distribution of output data at each pressure level. The Monte Carlo routine chose values for the benthic invertebrate group parameters from a uniform distribution with a mean equal to the estimates reported in the published model [35] and a coefficient of variation equal to 20%.

We compared the relative fits of a linear, piecewise, and sigmoidal model to the attribute-pressure relationships generated from each Monte Carlo data set using Akaike's information criterion corrected for small samples (AIC*_c_*; [38]). For each attribute, the model with the lowest AIC*_c_* value for the most data sets was selected as the best, and confidence intervals for the model parameters (and the utility threshold for nonlinear relationships) were obtained using a nonparametric bootstrap resampling procedure (*n* = 10,000 for each Monte Carlo data set). These analyses and those that follow were conducted using the nonlinear regression (nls) routine in R v2.8.1 [39] and the bbmle [40] package.

To illustrate the fourth step of the utility threshold analysis, we tested for correlations (Spearman rank, *r_s_*) between a single candidate indicator and the ecosystem attributes with significant utility thresholds in each Monte Carlo data set. In practice, however, a suite of indicators that describes a variety of ecosystem attributes should be used [18], [31]–[34]. We report the median value of the indicator-attribute correlations, along with their 95% confidence intervals. Similar to the attribute-pressure relationships, we compared the relative fits of linear, piecewise, exponential, and parabolic models (see Text S1 for details) to the relationships between the indicators and pressures generated from each Monte Carlo data set using AIC*_c_*. Confidence intervals for the model were obtained using a nonparametric bootstrap resampling procedure (*n* = 10,000 for each Monte Carlo data set).

We used spider plots to visualize trade-offs among the four ecosystem attributes and the two fisheries yield metrics under three different levels of anthropogenic pressure. The pressure levels corresponded approximately to a minimum-impact scenario in which none of the utility thresholds were breached, a threshold scenario in which the simulated pressure matched that of the lowest utility threshold, and a maximum-impact scenario representing the maximum pressure considered. In all plots the attributes and fisheries metrics were re-scaled so that the values were relative and fell within the interval [0,1], where zero corresponds to a stressed condition and one corresponds to an unstressed condition.

## Results

### Case study 1: Fishing pressure

Overfishing is a pervasive threat throughout the global ocean [41]. We ran a series of 50-year simulations in which fishing pressure for all commercially-targeted functional groups in the northern British Columbia ecosystem spanned zero to ten times the estimated baseline value (*n* = 15 simulations at 0, 0.2, 0.4, 0.6, 0.8, 1, 2, 3, 4, 5, 6, 7, 8, 9, 10 times the baseline fishing pressure). Fishing pressure was sustained at a constant level throughout each simulation. Deterministic simulations at each pressure level showed that all functional groups and ecosystem attributes had reached constant values by the end of 50 years, and these final values were used in the analyses that follow.

**Figure 2:**
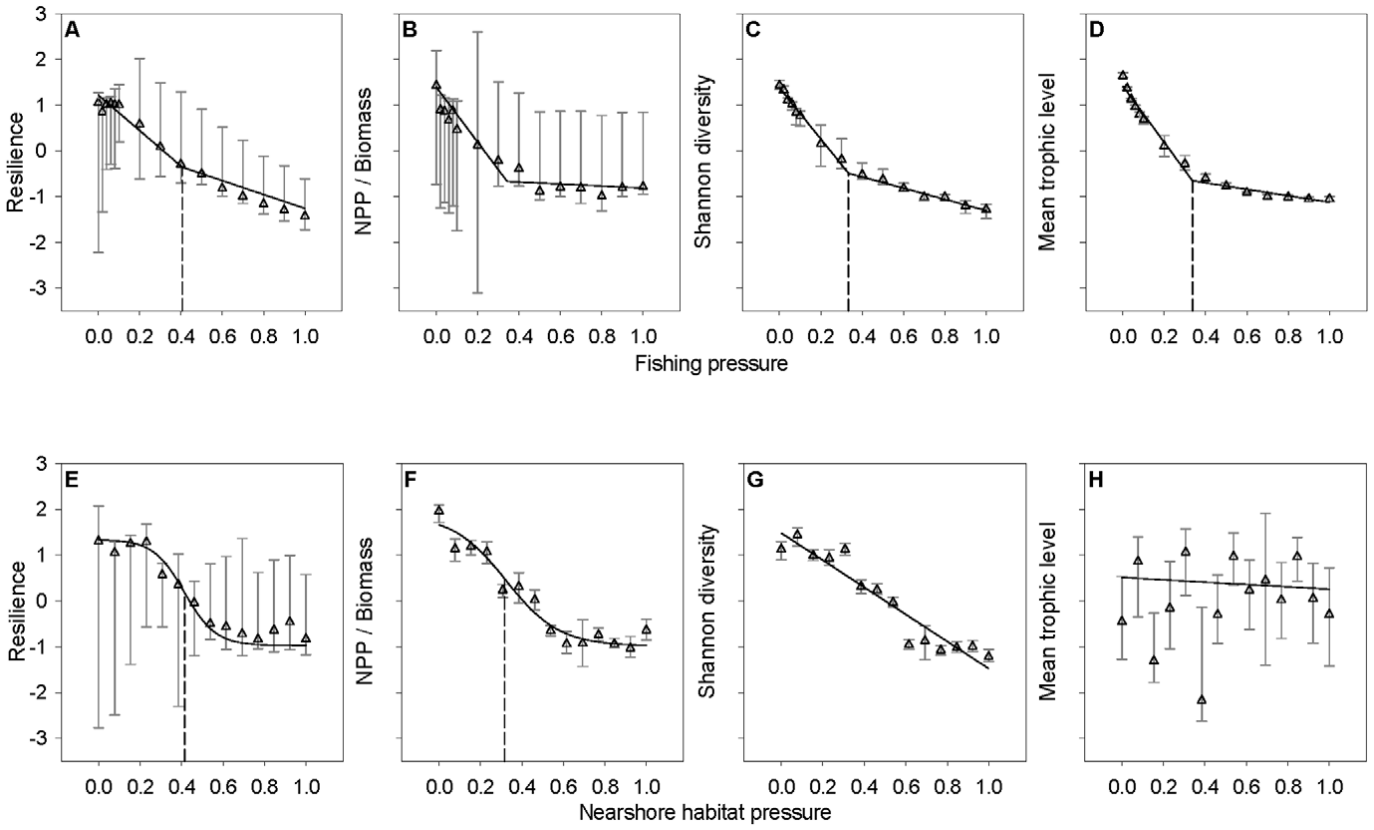
Model-generated relationships between 4 ecosystem attributes and increasing ecosystem-wide fishing (a-d) or nearshore habitat (e-h) pressure. The ecosystem attributes are resilience, NPP/Biomass, Shannon diversity, and mean trophic level. Open triangles indicate median values calculated from Monte Carlo simulated Ecopath with Ecosim data (*n* = 100), and error bars denote 95% confidence intervals. The solid lines represent best-fit functional relationships and the dotted lines designate significant utility thresholds estimated using a nonparametric bootstrap resampling procedure (*n* = 10,000 for each Monte Carlo data set) (parameter values and significant utility thresholds listed in Table 2). NPP = net primary production. In this and following figures, the ecosystem attributes (y-axes) have been re-scaled so that larger values are considered unstressed rather than stressed. The pressure values have been re-scaled relative to the maximum simulated pressure, and are contained within the range [0, 1].

Resilience, the NPP/Biomass ratio, Shannon diversity, and mean trophic level all declined with increasing fishing pressure (Fig. 2a-d). This finding is congruent with the hypothesized behavior of these ecosystem attributes [13], [42]–[45] and the effect of fishing on these attributes corroborates previous predictions made using the British Columbia model [35]. A piecewise function provided the best fit to the most Monte Carlo data sets for all four relationships (Table 2). Interestingly, the utility thresholds (median [95% confidence interval]) for resilience (0.41 [0.13, 0.66]), Shannon diversity (0.33 [0.13, 0.42]), and mean trophic level (0.34 [0.27, 0.40]) were relatively similar in magnitude. We did not interpret as significant the utility threshold for the NPP/Biomass versus fishing pressure relationship because the other three parameters in the best-fit model were statistically indistinguishable from zero (Table 2).

**Table 2:** Best-fit models and parameters for attribute–pressure relationships generated through fishing pressure (*n* = 15) and nearshore habitat pressure (*n* = 14) Monte Carlo simulations (*n* = 100 at each pressure level).

Attribute	Best-fit function	Model parameters
*Fishing pressure*		
Resilience	Piecewise (40/100)	*P_t_* = **0.41**, *b_1_* = 1.22, *m_1_* = −3.88, *m_2_* = **−1.51**
NPP / Biomass	Piecewise (37/100)	*P_t_* = **0.34**, *b_1_* = 1.42, *m_1_* = −6.15, *m_2_* = −0.71
Shannon diversity	Piecewise (93/100)	*P_t_* = **0.33**, *b_1_* = **1.37**, *m_1_* = **−5.58**, *m_2_* = **−1.22**
Mean trophic level	Piecewise (100/100)	*P_t_* = **0.34**, *b_1_* = **1.39**, *m_1_* = **−6.01**, *m_2_* = **−0.22**
*Nearshore habitat pressure*		
Resilience	Sigmoidal (46/100)	*c_0_* = **1.34**, *c_1_* = **0.007**, *c_2_* = **0.004**, *c_3_* = **13.39**, *P_t_* = **0.41**
NPP / Biomass	Sigmoidal (45/100)	*c_0_* = **1.87**, *c_1_* = **0.123**, *c_2_* = **0.081**, *c_3_* = **7.80**, *P_t_* = **0.32**
Shannon diversity	Linear (63/100)	*b_0_* = **1.48**, *m_0_* = **−2.95**
Mean trophic level	Linear (100/100)	*b_0_* = −0.25, *m_0_* = 0.51

For each attribute, linear, piecewise, and sigmoidal models were compared using AIC_*c*_. The model that was judged superior for the most Monte Carlo data sets was selected as the best-fit function and subjected to a nonparametric bootstrap procedure (*n* = 10,000 for each Monte Carlo data set) to determine parameter values and significance (**bold** indicates that 95% CI do not overlap zero). The units for the utility thresholds (*P_t_*) are relative, and are contained within the range [0, 1]. NPP = net primary production. See Text S1 for model and parameter definitions.

**Figure 3:**
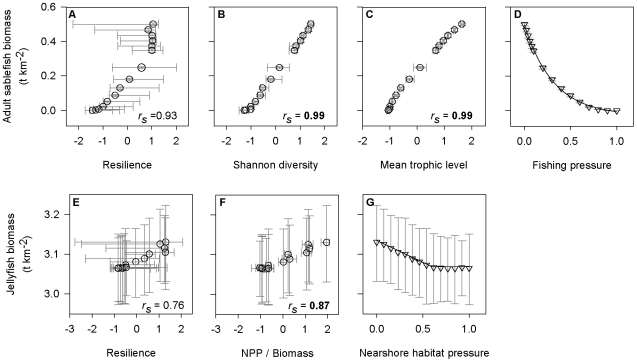
Correlations between indicators and attributes with significant utility thresholds, along with indicator-pressure relationships. The indicators are sablefish and jellyfish biomass for the fishing (a-d) and nearshore habitat (e-g) pressure simulations, respectively. Open circles (a-c, e-f) and triangles (d, g) indicate median values calculated from Monte Carlo simulated Ecopath with Ecosim data (*n* = 100 for each pressure level), and error bars denote 95% confidence intervals. The solid lines in (d) and (g) represent best-fit functional relationships estimated using a nonparametric bootstrap resampling procedure (*n* = 10,000 for each Monte Carlo data set) (parameter values listed in Table 3). *r_s_* = median spearman rank correlation across the Monte Carlo data sets (bold indicates 95% CI did not overlap zero), NPP = net primary production.

We chose to evaluate the potential for sablefish (*Anoplopoma fimbria*) to serve as an ecosystem indicator because this species is a valuable component of commercial and recreational fisheries, of conservation interest, amenable to conventional monitoring techniques, and already subject to regular stock assessments [46]. Across the 15 fishing pressure simulations, adult sablefish biomass strongly correlated with each of the 3 ecosystem attributes shown to have significant utility thresholds (resilience *r_s_* = 0.93 [−0.35, 0.99], Shannon diversity *r_s_* = 0.99 [0.98, 1], mean trophic level *r_s_* = 0.99 [0.99,1]; Figs. 3a-c). Though in practice sampling error or other sources of variation would likely reduce the strength of these model-generated correlations, and it would be more appropriate to analyze multiple indicators, for the purpose of illustration we focus only on adult sablefish biomass as a robust indicator of these ecosystem attributes.

**Table 3:** Best-fit models and parameters for indicator–pressure relationships generated through fishing (*n* = 15) and nearshore habitat pressure (*n* = 14) Monte Carlo simulations (*n* = 100 at each pressure level).

Pressure	Best-fit function for indicator	Model parameters	Utility threshold	Indicator value [95% CI]
Fishing	Neg. exponential (100/100)	*a_0_* = **-0.03**, *a_1_* = **0.52**, *a_2_* = −**3.15**	Shannon diversity	0.155 [0.147,0.163]
Nearshore habitat	Piecewise (71/100)	*P_t_* = **0.60**, *b_1_* = **3.13**, *m_1_* = **−0.11**, *m_2_* = 0.0003	NPP / Biomass	3.10 [3.00,3.18]

Indicator values corresponding to the utility thresholds for Shannon diversity and NPP/Biomass are shown for the fishing and nearshore habitat pressure simulations, respectively (also see *P_t_* values in Table 2). Linear, negative exponential, piecewise, and parabolic models were compared using AIC*_c_*. The model that was judged superior for the most Monte Carlo data sets was selected as the best-fit function and subjected to a nonparametric bootstrap procedure (*n* = 10,000 for each Monte Carlo data set) to determine parameter significance (**bold** indicates that 95% CI do not overlap zero). The value of the indicator (median ±95% CI) corresponding to the utility threshold was also derived from the Monte Carlo simulated data sets and bootstrap procedure. NPP = net primary production. See Text S1 for model and parameter definitions.

The exponential model provided the best fit to the sablefish-fishing pressure data (Table 3), and also showed good predictive value (*R^2^*>0.99; Fig. 3d). We used the bootstrapped parameter estimates and the exponential model (Table 3) to predict adult sablefish biomass at the median utility threshold for Shannon diversity (the lowest fishing pressure threshold: *P_t_* = 0.33). The rather precise estimate for the adult sablefish biomass (median [95% confidence interval]) corresponding to the Shannon diversity utility threshold was 0.155 t km^−2^ [0.147, 0.163] (Table 3).

**Figure 4:**
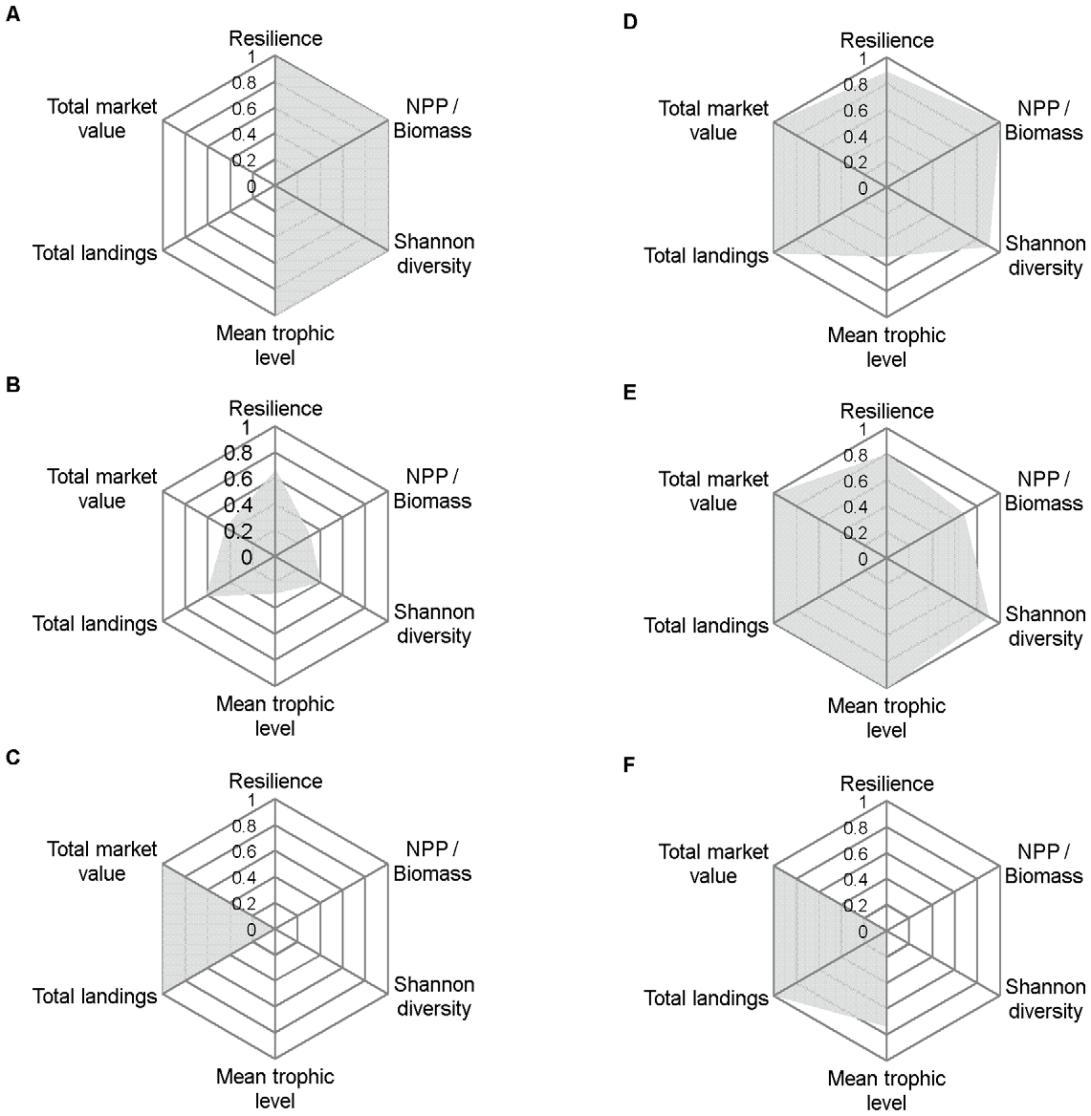
Spider plots depicting trade-offs among four ecosystem attributes and two fisheries yield metrics. The three different fishing (a-c) and nearshore habitat (d-f) pressure levels corresponded approximately to a minimum-impact scenario in which none of the utility thresholds were breached (a, d), a threshold scenario in which the simulated pressure matched that of the lowest utility threshold (median value; see Table 2) (b, e), and a maximum-impact scenario representing the maximum pressure considered (c, f). Note that for each type of pressure, all attributes have been re-scaled so that values are relative and fall within the interval [0,1], where zero corresponds to a stressed condition and one corresponds to an unstressed condition.

The trade-off analysis revealed that under the minimum-impact, no-fishing scenario, resilience, NPP/Biomass, Shannon diversity, and mean trophic level attained maximum values, while total landings and total market value of the fisheries were at minima (Fig. 4a). The four ecosystem attributes and the two fisheries yield metrics all reached 30–60% of their maximum values in the scenario corresponding to the lowest utility threshold (Shannon diversity; Fig. 4b). In the maximum fishing pressure scenario (ten times the baseline pressure), total landings and total market value of the fisheries attained maxima, whereas the 4 ecosystem attributes showed just the opposite response (Fig. 4c).

### Case study 2: Nearshore habitat pressure

Nearshore habitat pressure is ubiquitous throughout the world's coastal marine ecosystems [47]. The loss of canopy-forming algae along temperate rocky coastlines is particularly widespread, and is thought to be caused by a number of factors, including urbanization effects (such as eutrophication, sedimentation, and oil spills) and the overharvesting of sea urchin predators [48]–[51]. We ran a series of 50-year simulations in which we varied nearshore habitat pressure by altering the production forcing function for the “macrophytes” functional group in the northern British Columbia ecosystem model (primarily the kelps *Nereocystis leutkeana* and *Macrosystis integrifolia*). The pressure range we considered spanned zero to 1.3 times the estimated baseline value (n = 14 simulations at 0, 0.1, 0.2, 0.3, 0.4, 0.5, 0.6, 0.7, 0.8, 0.9, 1, 1.1, 1.2, and 1.3 times the baseline macrophyte production rate). In the analyses that follow, the highest nearshore habitat pressure corresponds to the lowest macrophyte production rate (zero times the baseline), while the lowest nearshore habitat pressure corresponds to the highest macrophyte production rate (a 30% increase over the baseline). As in the fishing pressure simulations, nearshore habitat pressure was sustained at a constant level throughout each model run. Deterministic simulations at each pressure level showed that all functional groups and ecosystem attributes had reached constant values by the end of 50 years, and these final values were used in the analyses that follow.

We used a simple linear mediation function to model the non-trophic, positive effect of macrophytes on several functional groups that use the structural complexity of these habitat-forming organisms as a refuge from predation. We included a strong mediation effect for juvenile herring, juvenile Pacific ocean perch, juvenile piscivorous rockfish, and juvenile planktivorous rockfish, a moderate mediation effect for inshore rockfish, and a weak mediation effect for adult lingcod, shallowwater benthic fish, and small crabs. The mediation functions caused the vulnerability of these groups to their predators to increase/decrease by up to 90% (strong), 50% (moderate), or 25% (weak) as macrophyte production declined/increased. In EwE models, vulnerability represents the maximum allowable increase in the predation mortality for each predator-prey interaction when predator biomass is high [52].

Resilience, the NPP/Biomass ratio, and Shannon diversity declined with increasing nearshore habitat pressure, whereas mean trophic level did not change in a consistent manner with nearshore habitat pressure (Figs. 2e–h). The sigmoidal model provided the best fit to the most Monte Carlo data sets for resilience and NPP/Biomass, whereas the linear model was judged best for Shannon diversity and mean trophic level (Table 2). The utility thresholds (median [95% confidence interval]) for the two attributes best-fit by a nonlinear model were relatively similar in magnitude, with the resilience threshold equal to a nearshore habitat pressure of 0.41 [0.34, 0.45]) and the NPP/Biomass threshold equal to a nearshore habitat pressure of 0.32 [0.07, 0.41] (Table 2).

In this case study, we chose to evaluate the potential for pelagic cnidarians and ctenophores (the carnivorous jellyfish group; hereafter, jellyfish) to serve as an ecosystem indicator because accumulating evidence suggests that human-induced pressures may be enabling their proliferation worldwide [53], [54]. Clearly, in a real application of this approach, testing of multiple indicators would be warranted in order to identify those that are most sensitive to changes in particular attributes, but for the sake of simplicity we focus on only one here. Across the 14 nearshore habitat pressure simulations, jellyfish biomass showed strong but variable Spearman rank correlations with the two ecosystem attributes characterized by significant utility thresholds (resilience *r_s_* = 0.76 [−0.96, 0.95], NPP/Biomass *r_s_* = 0.87 [0.76, 0.95]; Figs. 3e-f). Somewhat surprisingly, the correlations with these attributes were positive because jellyfish biomass, like resilience and NPP/Biomass, declined with increased nearshore habitat pressure (Fig. 3g). However, jellyfish are predominantly detritivores in this British Columbia model, and macrophytes contribute substantially to the detrital pool. Thus, increased nearshore habitat pressure caused a decline in a major component of the jellyfish diet, reducing their biomass.

At each level of nearshore habitat pressure, jellyfish biomass showed a considerable amount of variability (Fig. 3g). Nonetheless, we decided to treat jellyfish biomass as an ecosystem indicator in order to illustrate how such variability would influence an indicator's usefulness in detecting utility thresholds. The piecewise model provided the best fit to the jellyfish-nearshore habitat pressure data (Table 3), and showed good predictive value (*R^2^*>0.99; Fig. 3g). We used the bootstrapped parameter estimates and the piecewise model (Table 3) to predict jellyfish biomass at the median utility threshold for NPP/Biomass (the lowest nearshore habitat pressure threshold: *P_t_* = 0.32). The estimate for the jellyfish biomass (median [95% confidence interval]) corresponding to this threshold covered a wide range of values with a median equal to 3.10 t km^−2^ [3.00, 3.18] (Table 3).

In all three scenarios examined in the trade-off analysis, total landings and total market value of the fisheries were relatively unaffected (Figs. 4d-f). Under the minimum-impact scenario (in which macrophyte production was enhanced by 30% relative to the baseline), resilience, NPP/Biomass, and Shannon diversity attained maximum or near-maximum values, while mean trophic level reached an intermediate value (Fig. 4d). All four ecosystem attributes achieved 70–100% of their maximum values in the scenario corresponding to the lowest utility threshold (NPP/Biomass; Fig. 4e). In the maximum nearshore habitat pressure scenario (corresponding to no macrophyte production), the four ecosystem attributes were minimized (Fig. 4f).

## Discussion

One of the great challenges of transforming EBM from a philosophical approach to a set of executable management actions is the development of an appropriate toolkit [33], [55], [56]. Deciding which attributes to track in order to capture ecosystem-scale changes in status and contextualizing the values of measured attributes relative to desired ecosystem states and functions is fundamental to EBM [1], [4]. This context can be provided in a number of ways (e.g., by reference to historical baselines [41], [43], [50]), but here we introduce an approach based on utility thresholds.

We took advantage of the mathematical properties of nonlinear relationships between several ecosystem attributes and human-induced pressures to derive utility thresholds. As we use the term and define it mathematically, a utility threshold distinguishes modifications in pressure that have a large effect on an attribute's value from changes that have a much smaller influence [9]. For instance, in our case study of the northern British Columbia marine ecosystem, adjusting fishing pressure from 35% to 25% of the maximum produced a 20-fold greater improvement in Shannon diversity than a shift from 45% to 35% of the maximum pressure. In this example, policymakers might decide to set decision criteria so that management actions were sure to operate in the region of high return (in terms of attribute condition) on investment (in terms of changes in human-induced pressure), i.e., to the left of the utility thresholds identified in the attribute-pressure relationships. This interpretation presumes that the desired state of the ecosystem attributes is a less stressed condition, but in practice, that decision would be left to stakeholders and policymakers. Indeed, our analyses show that reducing fishing pressure causes clear sacrifices in fisheries yield. Such a trade-off may or may not be acceptable to decision makers. In other cases, as in the nearshore habitat pressure simulations, trade-offs will occur among ecosystem attributes but produce essentially no costs in terms of reduced fisheries yield. This outcome poses the difficult question of which ecosystem attributes are most important, but in so doing, it provides an explicit accounting of the impacts of alternative management actions.

As we have presented it, the utility threshold approach makes two key assumptions regarding ecosystem dynamics. First, it implicitly assumes that the qualitative form of an attribute-pressure relationship is stationary. However, in some systems this assumption may be invalidated by a change in the primary production regime or other environmental conditions (e.g., PDO, ENSO, etc.). Provided the effects of those changes on the ecosystem are understood sufficiently well (e.g., a climatic regime shift leads to predictable changes in species distributions, in turn affecting the relationship between diversity and a pressure; [57]), it would remain useful to identify multiple utility thresholds for each attribute-pressure relationship under alternative sets of environmental conditions. However, if such shifts are not anticipated, we caution that our method could provide misleading information about how reductions in human pressures will affect ecosystem attributes of concern.

The second crucial assumption of our approach is that the pathway to recovery for ecosystem attributes is the reverse of the one that created the stressed condition in the first place (i.e., there is no hysteresis). In the case study we presented, the EwE model did not predict hysteresis. However, because the basis of EBM is interactions between multiple human sectors [11], each of which causes pressure on an ecosystem, it is unlikely that the sequence and extent of management actions geared toward recovery will consistently mirror the pathway that led to the current state. Furthermore, a sound understanding of hysteresis exists in only a handful of cases (e.g., lakes, coral reefs; [23], [29]), suggesting a real need for additional empirical and theoretical study of ecosystem recovery pathways. Ecosystem models like EwE can be used to understand the disparate effects of increasing versus decreasing human pressures on ecosystem attributes if the hysteresis is caused by trophic interactions [58]. However, other modeling frameworks may be more appropriate in some cases (e.g., [23]), and such alternative models and/or empirical data will be required if hysteresis is caused by physical variables (e.g., nutrients; [29]). Prior to applying a utility threshold approach in other systems, we encourage researchers to quantify functional relationships for the effects of both increasing and decreasing pressure on ecosystem attributes [29].

Other aspects of the utility threshold analysis presented here are less critical to its interpretation. There are many nonlinear models besides the piecewise and sigmoidal functions we considered that might be used to determine the general shape of the attribute-pressure relationship [59]. Similarly, there are alternative mathematical definitions for utility thresholds that may be more appealing for some audiences than those presented here. For instance, a utility threshold could be defined as a point on an attribute-pressure curve distinguishing a region with slope equal to zero from a region with slope unequal to zero (e.g., similar to the Lowest Observed Adverse Effect Level (LOAEL) threshold used in ecotoxicology [25]). Any definition of the utility threshold point, using any set of nonlinear models, can be integrated with our overall method provided that the best-fit model and the threshold definition allow the objective identification of a point where the derivative of the attribute-pressure function shifts markedly.

Though we have outlined the utility threshold analysis procedure using one specific class of marine ecosystem model (EwE), the method is transferable to any other modeling framework. To be prescriptive in a specific ecosystem, our approach for quantifying utility thresholds should be implemented by examining empirical attribute-pressure, attribute-indicator, and indicator-pressure relationships (e.g., [5], [8], [15], [18], [22], [60], [61]) and generating multiple predictions about the form of such relationships using an ensemble of ecosystem models (e.g., in the same vein as climate projections produced by the Intergovernmental Panel on Climate Change). Multi-model inference techniques [38] can be used to achieve consensus about the functional form of attribute-pressure relationships, the precise location of utility thresholds, and indicator-pressure relationships. Similarly, it is particularly important to develop a suite of indicators that reliably tracks ecosystem attributes of interest in order to achieve confidence in the status of an ecosystem relative to utility thresholds [18], [31]–[34]. Finally, note that because we focus on attributes common to all ecosystems [13], [14], [16], [17], this approach is applicable to non-marine environments as well.

This type of analysis relies on a certain degree of subjectivity in the sense that stakeholders first must agree to focus their efforts on a potentially arbitrary set of attributes and pressures. However, public engagement and subjectivity are common to most conservation and management situations (e.g., [21], [62]) and, following these initial decisions, the approach we outline is entirely objective. It demonstrates quantitatively that all EBM actions are not created equal: the identification of utility thresholds reveals the relative ecological benefits of alternative policy decisions [6]. Further, the utility threshold approach provides a means to scientifically define, visualize, and operate along trade-offs among ecosystem attributes so that managers can make informed choices about the costs and benefits of alternative policies [2].

Our paper amplifies the notion that the answer to the question, “how much management action is enough?,” is “it depends”. It depends on management objectives and societal values since it is from these that the relative worth of different ecosystem attributes emerges [21], [33]. For any policy scenario, an understanding of utility thresholds helps to determine the amount of management intervention required to effect substantial improvement in various aspects of ecosystem structure and function. However, given conflicting demands on the ecosystem, it is up to policymakers to determine whether or not to breach a particular utility threshold in favor of some other objective. Ultimately, a thresholds-based approach lays bare the consequences of management actions, and we trust that such transparency will improve the ability of managers to protect and restore marine ecosystems.

## Supporting Information

Text S1 Models for attribute-pressure and indicator-pressure relationships, along with utility threshold definitions.
(0.07 MB DOC)
