# Supplementary material for: Identifying Thresholds for Ecosystem-Based Management
Source: PLoS One. 2010 Jan 26;5(1):e8907. doi: 10.1371/journal.pone.0008907 (PMC2811186; doi:10.1371/journal.pone.0008907)
Supplement: Text S1 — Models for attribute-pressure and indicator-pressure relationships, along with utility threshold definitions. (0.07 MB DOC) [file pone.0008907.s001.doc]

Text S1. Models for attribute-pressure and indicator-pressure relationships, along with utility threshold definitions.

# Models for attribute-pressure relationships and utility threshold definitions

Suppose that larger values of any ecosystem attribute *A* represent an unstressed condition, smaller values of *A* represent a stressed condition, and that increasing pressure *P* will cause a decline in *A*. Scaled in this way, we submit that many attribute-pressure relationships can be approximated by one of three different mathematical functions [1,2].

First, we define a linear decline in an attribute with increasing pressure (Fig. 1a) using the typical functional form

$A=b_{0}+m_{0}P+\varepsilon$ , (TS1-1)

where *b*_0_ is the *y*-intercept and *m*_0_  (-∞,0] is the slope of the line. In this model and those that follow ε are assumed to be independent, additive errors with zero mean and constant variance [3].

We also introduce two nonlinear models to describe attribute-pressure relationships. Though a variety of functions might have been exploited for this analysis, we chose the two below because they are simple, monotonic, and commonly-used. Most importantly, it is straightforward to define an objective threshold point using them.

The first nonlinear attribute-pressure relationship we describe is characterized by two lines joined at a breakpoint and has the functional form

$A=\left\{\begin{matrix} b_{1}+m_{1}P\text{ }+\varepsilon\text{ for }P\leq P_{t}\text{ } \\ b_{1}+m_{1}P_{t}+m_{2}(P-P_{t})\text{ }+\varepsilon\text{ for }P>P_{t} \end{matrix}\right.$ . (TS1-2)

In this piecewise model, *b*_1_ is the *y*-intercept, *m*_1_  (-∞,0] and *m*_2_  (-∞,0] are the slopes of the two lines, and *P_t_* is the breakpoint distinguishing the first line from the second. Similar functions have been used widely to identify thresholds in the ecotoxicological [4–6] and ecological [7–11] literatures. Note that this single functional form can accommodate two different shapes: concave-up (*m*_2_ > *m*_1_; Fig. 1b) and concave-down (*m*_1_ > *m*_2_; Fig. 1c). In our application, the utility threshold for an attribute-perturbation relationship fit using the piecewise model occurs at the point *P_t_* (indicated by the dashed lines in Figs. 1b-c).

The second nonlinear attribute-pressure relationship we describe is a sigmoidal function (Fig. 1d) with the form

$A=c_{0}\left(1-\frac{c_{1}}{c_{2}+e^{-c_{3}P}}\right)+\varepsilon$ . (TS1-3)

In this sigmoidal model, *c*_0_ is the primary determinant of the attribute’s value when the pressure is equal to zero, *c*_1_ describes the minimum decline in the value of the attribute as the pressure increases, and *c*_2_ and *c*_3_ together determine the steepness of the transition between large and small values of the attribute with increasing pressure. All *c_i_*  (-∞,∞).

Though there are many functions that can produce this sigmoidal shape, we chose to use equation (TS1-3) because it facilitates the use of an analytical definition for the utility threshold at the inflection point

$P_{t}=\frac{\text{log}\frac{1}{c_{2}}}{c_{3}}$ . (TS1-4)

This choice for the threshold is a natural one, as it marks the point where the function changes curvature (i.e., the second derivative equals zero). Modifying the amount of pressure near the threshold will produce much larger changes in the value of the attribute than will adjustments elsewhere. Theoretical discussions of ecological thresholds commonly refer to this type of attribute-pressure relationship, with the idea that the attribute will tend to diminish with increases in pressure beyond *P_t_* and will tend to rise with reductions in pressure below *P_t_* [2,12–14].

*Models for indicator-pressure relationships*

We tested the fit of linear, piecewise, exponential, and parabolic models to the relationship between the ecosystem indicator (adult sablefish or jellyfish biomass) and pressure (fishing or nearshore habitat) generated from each Monte Carlo data set (*n* = 100 pressure/level). We chose these models because they were simple, monotonic, and described the declines in the indicators in the northern British Columbia model output well. However, in other applications, it may be appropriate to consider a broader or different set of models in order to find one that predicts well changes in an ecosystem indicator *I* with increased pressure *P*.

The linear and piecewise models we used had the functional forms described in equations (TS1-1, TS1-2). The exponential model had the form

$I=a_{0}+a_{1}e^{(a_{2}P)}$ , (TS1-5)

while the parabolic model had the form

$I=b_{2}+m_{3}P+m_{4}P^{2}$ . (TS1-6)

We calculated the proportion of variation explained by the best-fit model for the indicator-attribute relationship generated by each Monte Carlo simulation using the nonlinear approximation for *R*^2^

$R^{2}=1-\frac{\text{SS}_{\text{reg}}}{\text{SS}_{\text{tot}}}$ , (TS1-7)

where SS_reg_ is the residual sum of squares given the model and SS_tot_ is the total sum of squares in the response. We report the median *R*^2^ value for the best-fit model across the 100 Monte Carlo data sets for each case study. All analyses were conducted using the nonlinear regression (nls) routine in R v2.8.1 [15] and the bbmle package [16].

References

1. Scheffer M, Brock W, Westley F (2000) Socioeconomic mechanisms preventing optimum use of ecosystem services: an interdisciplinary theoretical analysis. Ecosystems 3: 451-471.

2. Andersen T, Carstensen J, Hernández-García E, Duarte CM (2009) Ecological thresholds and regime shifts: approaches to identification. Trends in Ecology & Evolution 24: 49-57.

3. Toms JD, Lesperance ML (2003) Piecewise regression: a tool for identifying ecological thresholds. Ecology 84: 2034-2041.

4. Yanagimoto T, Yamamoto E (1979) Estimation of safe doses: critical review of the hockey stick regression method. Environmental Health Perspectives 32: 193-199.

5. Horness BH, Lomax DP, Johnson LL, Myers MS, Pierce SM et al. (1998) Sediment quality thresholds: estimates from hockey stick regression of liver lesion prevalence in English sole (P*leuronectes vetulus*). Environmental Toxicology and Chemistry 17: 872-882.

6. Suter, GW (2007) Ecological risk assessment. Boca Raton, FL: CRC Press.

7. Framstad E, Stenseth NC, Bjoernstad ON, Falck W (1997) Limit cycles in Norwegian lemmings: tensions between phase-dependence and density-dependence. Proceedings of the Royal Society B: Biological Sciences 264: 31-38.

8. Barrowman NJ, Myers RA (2000) Still more spawner-recruitment curves: the hockey stick and its generalizations. Can J Fish Aquat Sci 57: 665-676.

9. Toms JD, Lesperance ML (2003) Piecewise regression: a tool for identifying ecological thresholds. Ecology 84: 2034-2041.

10. Lindenmayer DB, Fischer J, Cunningham RB (2005) Native vegetation cover thresholds associated with species responses. Biological Conservation 124: 311-316.

11. Samhouri JF, Vance RR, Forrester GE, Steele MA (2009) Musical chairs mortality functions: density-dependent deaths caused by competition for unguarded refuges. Oecologia 160: 257-265.

12. Scheffer M, Brock W, Westley F (2000) Socioeconomic mechanisms preventing optimum use of ecosystem services: an interdisciplinary theoretical analysis. Ecosystems 3: 451-471.

13. Scheffer M, Carpenter S, Foley JA, Folke C, Walker B (2001) Catastrophic shifts in ecosystems. Nature 413: 591-596.

14. Suding KN, Hobbs RJ (2009) Threshold models in restoration and conservation: a developing framework. Trends in Ecology & Evolution 24: 271-279.

15. R Development Core Team (2009) R: A language and environment for statistical computing. Vienna, Austria: R Foundation for Statistical Computing.

16. Bolker B (2008) bbmle: Tools for general maximum likelihood estimation. R package version 0 8 5 based on stats4 by the R Development Core Team .
